# Challenges and outcomes of haemodialysis among patients presenting with kidney diseases in Dodoma, Tanzania

**DOI:** 10.1186/s12882-017-0634-2

**Published:** 2017-07-04

**Authors:** Alfred J. Meremo, David P. Ngilangwa, Masumbuko Y. Mwashambwa, Matobogolo B. Masalu, Janet Kapinga, Rehema Tagalile, Issa Sabi

**Affiliations:** 1 School of Medicine & Dentistry, College of Health Sciences, The University of Dodoma, P.O Box 395, Dodoma, Tanzania; 2 Haemodialysis Unit, The University of Dodoma, P.O Box 259, Dodoma, Tanzania; 3 Amref Health Africa, P.O Box 2773, Dar es Salaam, Tanzania; 4 National Institute for Medical Research, Mbeya Medical Research Centre, P.O Box 2410, Mbeya, Tanzania

**Keywords:** Challenges, Outcomes, Haemodialysis, Chronic kidney diseases (CKD), Tanzania

## Abstract

**Background:**

Kidney Diseases contribute a significant proportion to the global burden of non-communicable diseases. Haemodialysis services as the main modality of renal replacement therapy in most resource limited countries is only available in few cities and at higher costs. The aim of this study was to determine the challenges and outcomes of patients who were on haemodialysis at the University of Dodoma (UDOM) haemodialysis unit in Tanzania.

**Methods:**

In this retrospective study; we reviewed haemodialysis registers and charts of 116 patients dialyzed from January 2013 to June 2015 at The UDOM haemodialysis unit. Data were descriptively and inferentially analysed using Stata version 11 software.

**Results:**

Of the 116 patients, 52 (44.9%) were male, and 38(32.8%) were married. Their median age was 45 years. Thirty-two (27.6%) had acute kidney injury, of them 26 (81.3%) patients had recovery of renal function after haemodialysis. Indications for hemodialysis were anuria (18), intoxications (14), electrolyte imbalance (9), uraemia (7) infections (6) and fluid overload (4). Eighty-four (72.4%) patients had End Stage Renal Diseases (ESRD), of which 37 (44.1%) absconded/lost to follow up, 15 (17.9%) died, 22 (26.2%) were referred to Muhimbili National Hospital (MNH), 12 for possible kidney transplant abroad after haemodialysis, and 10 (11.9%) were still attending our unit for haemodialysis. Residing outside Dodoma was predictive for poor outcomes while on haemodialysis (OR 5.2, 95% CI 3.2–8.6, *p* < 0.001). In addition the odds ratio for poor outcomes was 7.3 times for a patient ESRD (OR7.34, 95% CI 3.26–18.17, *p* < 0.001). Patients who had no National Health Insurance Fund (NHIF) coverage (OR 6.6, 95% CI 5.4–12.7, *p* < 0.001) also had higher odds of poor outcomes after starting haemodialysis.

**Conclusion:**

Unavailability and high costs related to utilization of haemodialysis services among patients needing dialysis are the challenges for better outcomes. Therefore, haemodialysis and renal transplants services should be made easily available in regional referral hospitals at reasonable costs. In addition, members of the public should be educated on joining health insurance schemes and on making healthy life style choices for preventing chronic kidney disease and its progression.

## Background

Kidney Diseases contributes a significant proportion to the global burden of Non-Communicable Diseases (NCDs) [1, 2]. These diseases together with other chronic Non-Communicable Diseases (NCDs) like diabetes, hypertension, malignancies, and cardiovascular diseases are rapidly increasing, particularly in developing countries [3]. In 2008 studies reported that 1.75 million patients worldwide received dialysis, of which 1.55 million (89%) were on haemodialysis (HD), nearly 62% of the HD patients were being treated in high-income countries and the remaining 38% in low and middle-income countries [4–6]. A systematic Medline search for observational studies and renal registries conducted in 2010 reported that at least 2·284 million people might have died prematurely because RRT could not be accessed. The largest treatment gaps were noted in low-income countries particularly Asia and Africa [7].

A systematic review conducted between 1998 and March 2013 showed that economic evaluation of RRT in low and middle-income countries faces methodological challenges, clearly indicating that the costs are beyond the capability of the average individual [8]. Most developed countries can provide peritoneal dialysis (PD) at a lesser expense as compared to HD, the evidence from developing countries is more mixed, but in most cases PD can be provided at a similar cost to HD where economies of scale have been achieved, either by local production or by low import duties on PD equipment [9, 10]. These programs can be sustainable only when governments can afford to support them by allocating funds in their national health budgets [11]. Studies conducted in Nigeria showed that challenges encountered were a combination of several factors namely: late presentation, co morbid conditions, location of the renal care centers, and inability to pay for the recommended adequate dialysis owing to high cost [12, 13]. Sustainability of maintenance haemodialysis is poor in most developing countries and kidney transplantation, which is cost-effective in the long-term, is rarely performed [14].

End Stage Renal Disease (ESRD) has been increasing; it is a major issue of public health concern currently. People in Sub Saharan Africa and other developing countries are at higher risk [2, 15]. Studies report that management of patients with ESRD in low and middle-income countries to be too expensive that most governments are un able to afford, resources and budgets that are allocated are unable to meet the burden of treatment [16]. The magnitude of ESRD in Tanzania is not known as there are no national registries for this disease. However, studies have reported a higher prevalence of CKD among diabetes mellitus patients attending clinic at Bugando Medical Centre in Mwanza [17]. Renal transplantation is not performed in Tanzania and only a few patients can afford going abroad. Thus, haemodialysis has been the main form of RRT available in Tanzania, very few centers offer peritoneal dialysis. Dialysis was offered at three public and nine private hospitals, 75% of all dialysis providers were concentrated in Dar es Salaam, the largest commercial city of the country [8, 18].

Furthermore, majority of patients are poor and are un able to access haemodialysis services in Tanzania. Only few people have enrolled into health insurances schemes such as National Health Insurance Fund (NHIF) or other private-owned insurance schemes, most patients with ESRD would have to pay by themselves for dialysis services [8, 18]. Limited data is available on the challenges of haemodialysis in Tanzania; therefore the aim of this study was to identify challenges and outcomes among patients presenting for haemodialysis services in Dodoma, Tanzania.

## Methods

### Study design, population and settings

This was a retrospective study; data was collected from 116 patients aged between 10 and 79 years old who were dialyzed at haemodialysis unit of The University of Dodoma (UDOM) in central Tanzania from January 2013 to June 2015. UDOM launched its haemodialysis unit on January 2013 as the first and only dialysis unit serving as referral centre for six regions namely Dodoma, Singida, Morogoro, Iringa, Manyara, and Tabora. It also serves other regional referral hospitals in the central zone and other neighbouring zones, serving a total population of 20 million people. The unit has a total of 10 haemodialysis machines, 3 physicians and 3 medical officers trained on haemodialysis, 6 dialysis nurses, 2 biomedical engineers, 2 laboratory technicians, 2 social workers and 1 nutritionist. Each dialysis session was charged at a cost of $ 150 for those on NHIF scheme and $ 175 for those paying out of pocket. The NHIF insurance scheme covered all patients who are insured to the scheme irrespective of whether they presented with acute kidney injury (AKI) or ESRD. All patients who required dialysis were dialyzed. Patients who had no NHIF insurance scheme were started on haemodialysis after arrangements were made with the hospital on how to settle their bills while on treatment.

### Data collection and laboratory procedures

Haemodialysis registers and charts of 116 patients dialyzed from January 2013 to June 2015 were reviewed. Information on patient socio-demographics, clinical characteristics, and relevant laboratory investigations were extracted. The study team reviewed haemodialysis charts for the number of sessions, duration of hemodialysis and outcomes of patients on haemodialysis. Estimated Glomerular Filtration Rate (eGFR) was calculated using the Chronic Kidney Disease Epidemiology Collaboration (CKD-EPI) equation. The Bedside IDMS traceable Schwartz GFR Calculator for Children was used to stage patients and ESRD was established according to Kidney Disease Improving Global Outcomes guidelines. The diagnoses were based on established clinical criteria [18]. AKI was defined as an acute deterioration in renal excretory function, with a serum urea >10 mmol/l and/or a rise in serum creatinine (Scr) by ≥0.3 mg/dl, or a percentage increase in Scr of ≥50% from baseline using the Acute Kidney Injury Network(AKIN) criteria [19]. AKI patients who required dialysis presented with an acute deterioration in renal excretory function and had indications for dialysis (anuria, electrolyte imbalance, fluid overload and uremia). ESRD had progressive chronic kidney disease with eGFR ≤15 mL/min/1.73 m2 with/without other indications for haemodialysis. ESRD was diagnosed in patients who had a progressive chronic kidney disease with eGFR ≤15 mL/min/1.73 m2, patients who had normochromic normocytic anaemia and findings on ultrasound that had features suggestive of chronic kidney disease. Outcome measures in those patients who absconded/lost to follow up and those patients who died after starting haemodialysis were not included. Patients gave informed consent and ethical approval was obtained before starting haemodialysis.

The UDOM haemodialysis unit utilizes both paper-based and electronic medical records which allow recording collection of patients’ health related activities in real-time. The study data were obtained from handwritten medical records and then cross-checked with electronic ones. Any discrepancies were reviewed and verified to ensure the validity of data thus data completeness is the strength of this study.

### Data management and analysis

Data collected were entered into a computer using epidata version 3.1 (CDC, Atlanta, USA) and analyzed using STATA version 11 (College Station, Texas, USA). Data were summarized in form of proportions and frequency tables for categorical variables. Depending on variable distribution, either mean with standard deviation or median with interquartile range were used to summarize continuous data. The correlation between getting poor outcomes based on different patient parameters were determined by performing logistic regression analyses. Odds ratios (OR) were calculated to estimate the percentage change of risk of poor outcomes while on haemodialysis. Parameters with *p*-values <0.05 were considered statistically significant.

## Results

### Socio-demographic and clinical characteristics of the study population

A total of 116 patients received haemodialysis during study period, of which 52 (44.9%) were male, and 38(32.8%) were married. The median age was 45 years (Interquartile Range (IQR) 10–79), 12 (10.4%) and 4 (5.2%) patients had HIV and Hepatitis B infections respectively; and no patient had hepatitis C infection. Glomerular filtration rate: < 15 mL/min/1.73 m2 accounted for 72.4% of the patients on haemodialysis (Table 1). A total of 5220 dialysis sessions were conducted during this period, most patients with AKI had about 8–10 sessions before recovery, with a range of 4–16 sessions. A total of 14 patients with AKI and 25 patients on ESRD were on the NHIF scheme.

**Table 1:** Baseline characteristics of 116 patients who got haemodialysis at The University of Dodoma haemodialysis unit

Charactericts	Proportion (%) or Median
Sex	
Male	52 [44.9%]
Age in years	45 [10–79]
Residency	
Dodoma	46 [39.7%]
Outside Dodoma	70 [60.3%]
Marital status	
Never Married	21 [18.1%]
Married	38 [32.8%]
Divorced	30 [25.9%]
Widow	27 [23.2%]
Haemoglobin (g/dl)	5.2 [3.8 - 12.6]
Serum creatinine level (μmol/L)	592 [121–1367]
Urea level (mmol/L)	26[7–53]
HIV positive Status	12 [10.4%]
Hepatitis B positive	4 [5.2%]
Type of Kidney Disease	
AKI	32 [27.6%]
ESRD	84 [72.4%]
NHIF	39 [33.6%]

### Causes of kidney diseases

Of the 116 patients 32 (27.6%) were found to have AKI, indications for hemodialysis were anuria (18), intoxications (14), electrolyte imbalance (9), uraemia (7) infections (6) and fluid overload (4). Intoxication (herbs and drugs) was the leading cause of acute kidney injury with 14 (43.8%) patients. Other causes included Eclampsia in 7 (21.8%) patients, Infections (Malaria, bacteria) in 6 (18.8%) patients and Post partum haemorrhage in 5 (15.6%) patients. A total of 26/ 32 (81.3%) patients had their renal recovery. They all recovered their renal function fully and after 6 months follow up exams showed their renal excretory function was normal.

Eighty-four (92.4%) patients had ESRD**,** of which 34(40.5%) had hypertension, 19(22.5%) had Diabetes Mellitus, 15(17.9%) had Chronic glomerulonephritis, 12(14.3%) had HIV/AIDS and 4(4.8%) had Hepatitis. A total of 15(17.9%) patients with ESRD died (Table 2). Most patients with ESRD presented with electrolyte imbalance, fluid overload and uremia.

**Table 2:** Causes of kidney diseases for 116 patients who had haemodialysis

Cause of Kidney disease AKI (*n* = 32)	Number (%)	Number of Deaths
Intoxication (herbs and drugs)	14 (43.8%)	1 died
Eclampsia	7 (21.8%)	3 died (DIC)
Infections (Sepsis) (Malaria, bacteria)	6 (18.8%)	2 died (septic shock)
Postpartum haemorrhage	5 (15.6%)	All recovered
ESRD (*n* = 84)	Number (%)	
Hypertension	34 (40.5%)	5 died
Diabetes Mellitus	19 (22.5%)	3 died
Chronic glomerulonephritis	15 (17.9%)	2 died
HIV/AIDS	12 (14.3%)	3 died
Hepatitis	4 (4.8%)	2 died

### Outcomes of patients who were on Haemodialysis

Of the 84 patients who had ESRD and haemodialysis, 37 (44.1%) patients absconded, could not afford the costs, or were lost to follow up. Most of these patients came from the nearby regions and zones. A total of 22 (26.2%) patients with ESRD and on dialysis were referred to Muhimbili National Hospital (MNH), 12 for renal transplants abroad and others for further management. A total of 15(17.9%) patients with ESRD died while on haemodialysis and 10 (11.9%) patients with ESRD were continuing with haemodialysis. Most patients with ESRD died because of cardiovascular complications and few had sepsis. Most patients with ESRD had about 5–8 haemodialysis sessions before absconding, referral or death.

### Predictors of poor outcomes while on haemodialysis

A total of 15 predictors of poor outcomes while on HD were identified after performing univariate logistic regression analyses. Backward elimination reduced this to 5 parameters. The potential predictors identified were gender, type of kidney disease, residence of patients and NHIF status of patients. Gender and age were found to be significant in univariate logistic regression analyses. However, this significance was lost after multivariate logistic regression analyses (Table 3).

**Table 3:** Adjusted odds ratios of poor outcomes

Parameters	Adjusted OR (95% confidence interval)	*P*-value
Gender		
Male	1.00	0.494
Female	1.1 (0.9–2.0)	
Age		
< 40 years	1.00	0.148
Above 40 years	1.3 (0.8–2.6)	
Residency		
Dodoma	1.00	<0.001
Outside Dodoma	5.2 (3.2–8.6)	
NHIF status		
YES	1.00	<0.001
NO	6.6 (5.4–12.7)	
Type of Kidney Disease		
ESRD	7.34 (3.26–18.17)	<0.001
AKI	0.56 (0.48–1.88)	0.657

Residing outside Dodoma was predictive of poor outcomes while on haemodialysis (OR 5.2, 95% Confidence Interval (CI) 3.2–8.6, *p* < 0.001). In addition, the multivariate adjusted odds of poor outcomes while on haemodialysis were 7.3 times higher for a patient with ESRD (OR7.34, 95% CI 3.26–18.17, *p* < 0.001). Patients who had no NHIF coverage (OR 6.6, 95% CI 5.4–12.7, *p* < 0.001) also had higher odds of poor outcomes

## Discussion

This study evaluated challenges and outcomes of patients on haemodialysis in Dodoma, Tanzania. Of the 84 patients who had ESRD and were started on haemodialysis, 37 (44.1%) patients absconded/lost to follow up and most of them were residing outside Dodoma. Probably this was due the difficulties of establishing a new life away from their native homes. This was a predictor of poor outcomes while on haemodialysis (OR 5.2, 95% CI 3.2–8.6, *p* < 0.001). Similar findings have been reported from other studies; in a study conducted at MNH reported that majority of Tanzanians had no access to haemodialysis services because the services were only available in few cities [18]. In other studies it was reported that only 38% of patients in low and middle-income countries regularly receive RRT in the form of haemodialysis [4–7]. Studies from Nigeria and Sub Saharan Africa reported that challenges encountered by patients on haemodialysis were a combination of several factors including location of the renal care centres and inability to pay for the recommended adequate dialysis owing to high cost [12, 13].

Of the 37 (44.1%) patients who absconded/ lost to follow up most of them could not afford the costs. 77(66.4%) patients were paying out of their pockets for haemodialysis. Patients who were not on NHIF scheme had higher odds of poor outcomes. This finding is similar to other studies; a study conducted between 1998 and 2013 showed that haemodialysis in low and middle-income countries were faced with severe financial barriers to access dialysis services and dialysis patients could not afford dialysis on the free market [8, 18]. Studies from Nigeria and Sub Saharan Africa reported that many patients were unable to pay for the recommended adequate dialysis owing to high costs; only 6.8% of patients were able to afford haemodialysis beyond 3 months [12–14]. Poor accessibility to proper management of ESRD in low and middle-income countries has been precipitated by inadequate allocation of funds by governments to meet the costs of treatment [16].

In this study we found that a total of 22 (26.2%) patients with ESRD and on dialysis were referred to MNH in Dar es Salaam for possible kidney transplants abroad. This finding is consistence with other studies whereby poor sustainability of maintenance haemodialysis services was observed and efforts to improve other modalities of RRT, in particular kidney transplantation which is cost-effective in the long-term were recommended [14, 20, 21].

Glomerular filtration rate: < 15 mL/min/1.73m^2^ accounted for 72.4% of the patients on haemodialysis and 15(17.9%) patients with ESRD died while on haemodialysis. Poor outcomes while on haemodialysis were 7.3 times higher for a patient with ESRD (OR7.34, 95% CI 3.26–18.17, *p* < 0.001). Many patients with ESRD had their outcomes not known either because they were absconded (37/84) or were referred to MNH (22/84) which probably have contributed to the observed low mortality. Only 25 patients had their outcome known and if we included only these patients in the analysis (15/25) the mortality among patients with ESRD will go up 60% which probably is the reflection of the real situation.

A study in Tanzania reported that hypertension related diseases were the most common cause of hospital admissions and accounted for most deaths [22]. In this study, of the 84 patients with ESRD, 40.5% (34/84) had hypertension (the leading condition) and 5 of them died. Similar findings were reported in Nigeria whereby long standing hypertension was seen in 70 (38.8%) patients with ESRD [23]. In contrast, other studies done in Nigeria and sub Saharan Africa reported chronic glomerulonephritis as the leading causes of ESRD [12, 24, 25] . This may reflect lifestyle differences, geographical variability and a greater control of infectious diseases.

Of the 116 patients who were dialyzed 32(27.6%) were found to have AKI. Intoxications were the leading cause of AKI accounting for 14(43.8%) patients. These findings are different from other studies; a study conducted in Nigeria reported that AKI constituted 14.8% of diagnoses of patients on haemodialysis [14]. Another study conducted in Nigeria showed the leading causes of AKI were Pre-eclampsia/eclampsia and drug nephrotoxicity [12]. Also in another study, sepsis was the leading cause of AKI [23]. These differences could be accounted for by the differences in the geographical and study populations. A total of 6/32 (18.7%) patients of those with AKI died. Disseminated Intravascular Coagulopathy (DIC) and complicating sepsis were the leading causes of death among these patients, similar finding have been reported from other studies [12, 23].

### Study limitations and strengths

This study was conducted in a haemodialysis unit in central part of Tanzania and as such the results are limited to patients who had ESRD and AKI in the central part of Tanzania. Secondly, being a referral center means only patients who were identified and were able to afford transport and treatment costs away from their home were included in this study, therefore findings may not be a true reflection of the true burden in the community. However, this information will provide insight to strategies for improving haemodialysis services elsewhere in Tanzania.

## Conclusions

Haemodialysis services should be made easily available in Tanzania at regional referral hospitals at reasonable costs that clients can afford. The public should be encouraged to join the NHIF. Health education and promotion to the general public should be highly emphasized and encouraged regarding use of local herbs, routine screening and checking blood pressures and blood sugars, and compliance with medications as a preventive strategy against kidney diseases. It’s time to start performing renal transplants within the country in order to provide exit points to patients on chronic haemodialysis.

## Abbreviations

AKI: acute kidney injury
AKIN: acute kidney injury network
CKD: chronic kidney diseases
CKD-EPI: chronic kidney disease epidemiology collaboration
eGFR: estimated glomerular filtration rate
ESRD: end stage renal disease
IDMS: isotope dilution mass spectroscopy
KDIGO: kidney disease improving global outcomes
MNH: muhimbili national hospital
NCDs: non communicable diseases
NHIF: national health insurance fund
UDOM: University of Dodoma.
